# Coordinated proteome change precedes cell lysis and death in a mat-forming cyanobacterium

**DOI:** 10.1038/s41396-023-01545-3

**Published:** 2023-11-01

**Authors:** Jackie Zorz, Alexandre J. Paquette, Timber Gillis, Angela Kouris, Varada Khot, Cigdem Demirkaya, Hector De La Hoz Siegler, Marc Strous, Agasteswar Vadlamani

**Affiliations:** 1 Department of Earth, Energy, and Environment, University of Calgary, Calgary, AB Canada; 2 Synergia Biotech Inc., Calgary, AB Canada; 3 Department of Chemical and Petroleum Engineering, University of Calgary, Calgary, AB Canada

**Keywords:** Metagenomics, Water microbiology, Proteomics, Microbial ecology

## Abstract

Cyanobacteria form dense multicellular communities that experience transient conditions in terms of access to light and oxygen. These systems are productive but also undergo substantial biomass turnover through cell death, supplementing heightened heterotrophic respiration. Here we use metagenomics and metaproteomics to survey the molecular response of a mat-forming cyanobacterium undergoing mass cell lysis after exposure to dark and anoxic conditions. A lack of evidence for viral, bacterial, or eukaryotic antagonism contradicts commonly held beliefs on the causative agent for cyanobacterial death during dense growth. Instead, proteogenomics data indicated that lysis likely resulted from a genetically programmed response triggered by a failure to maintain osmotic pressure in the wake of severe energy limitation. Cyanobacterial DNA was rapidly degraded, yet cyanobacterial proteins remained abundant. A subset of proteins, including enzymes involved in amino acid metabolism, peptidases, toxin-antitoxin systems, and a potentially self-targeting CRISPR-Cas system, were upregulated upon lysis, indicating possible involvement in the programmed cell death response. We propose this natural form of cell death could provide new pathways for controlling harmful algal blooms and for sustainable bioproduct production.

## Introduction

Eutrophication, resulting in expansion and prolongation of cyanobacterial mats and blooms, is expected to increase in frequency and severity with climate change and continuing industrialization [1, 2]. Disintegration of these mats and blooms can lead to increased heterotrophic respiration causing suffocation of aquatic life, and release of potent cyanotoxins that have adverse health effects for humans and animals [3]. Similarly, cyanobacteria grown for biotechnological purposes experience periodic mass culture crashes that negatively impact commercial operations [4]. Despite the economic and environmental importance, little is known about the cause leading to the breakdown of dense cyanobacterial growth and the molecular response of cyanobacterial cells prior to their death [5, 6].

The potential causes of cyanobacterial death include viral attack, predation, and programmed cell death [7, 8]. Cyanobacterial viruses (cyanophages) and eukaryotic grazers are known to control cyanobacterial dynamics in natural freshwater and marine environments and have also been implicated in mass die-offs of cultures and blooms [9, 10]. Recently, however, more attention has been paid to the potential importance of programmed cell death in defining cyanobacterial dynamics in nature as a response to both biotic and abiotic threats [8, 11, 12]. Programmed cell death, defined as genetically encoded processes leading to cellular suicide [13], in single-celled prokaryotes, would appear to be an evolutionary paradox. However, evidence for this altruistic behavior has been observed in several situations, including thwarting viral propagation, and in multicellular communities, providing nutrients and metabolites to “persister” cells, which are members of a genetically analogous population that do not undergo cell death but rather persevere until conditions improve, ultimately enhancing inclusive fitness [14–16].

Programmed cell death has been considerably less studied in prokaryotes compared to eukaryotes, but some hallmarks, including DNA fragmentation, protein degradation, breakdown of ribosomes and RNA, and the involvement of reactive oxygen species are known [13, 14]. Studies have also implicated toxin-antitoxin systems and CRISPR-Cas systems as potential mechanisms in prokaryotic programmed cell death. Toxin-antitoxin systems are widespread protein or RNA-encoding genetic elements, that contain a toxin capable of inhibiting cell growth, and an antitoxin that counteracts the toxin [17]. Toxin-antitoxin systems often exert their toxic effects by targeting protein translation or DNA replication [17]. CRISPR-Cas systems, specifically Type III systems, have also been implicated in programmed cell death in prokaryotes, mainly observed to act as indiscriminate nucleases during abortive infection, whereby infected cells altruistically commit suicide to prevent further spread of an infectious agent [18–20]. Despite these observations, global molecular surveys of genes involved in prokaryotic programmed cell death are lacking, so the exact cellular components and proteins taking part in this process are largely unknown [6].

To investigate the process of cell death in cyanobacteria, we used an alkaliphilic cyanobacterial consortium that was observed, during preliminary experiments, to undergo a mass lytic event midway through a 12-day dark and anoxic incubation. This cyanobacterial consortium was previously isolated from the productive benthic mats of an alkaline (pH >10, alkalinity >0.5 M) soda lake [21, 22], and consists of the cyanobacterial species “*Candidatus* Phormidium alkaliphilum” [23] (renamed to *Sodalinema alkaliphilum* in GTDB version 214) in association with several other microbial members [24]. The dark and anoxic incubation could be considered similar to the conditions that the cyanobacteria would experience in nature with a cessation of photosynthesis and heightened respiration brought about by nighttime or through sinking into the aphotic regions of microbial mats or lake sediments [25–27]. In the natural soda lake environment, the prolific growth of cyanobacteria in these mats does not appear to translate into a build-up of biomass or sediment. Instead, the presence of steep oxygen and sulfide gradients within the mats and lake sediments, indicates that cyanobacteria are rapidly turned over [25–27].

We monitored the lysis of cyanobacterial cells by performing metagenomics and metaproteomics on samples collected throughout a 12-day dark and anoxic incubation of the consortium. From this data, there was no evidence for ecological interactions such as predation by other bacteria, eukaryotes, or viruses as the cause of cell lysis. Instead, proteogenomic data suggested that lysis likely resulted from programmed cell death provoked by energy starvation. We propose that mass die-offs and culture crashes commonly associated with dense growth of certain cyanobacterial taxa could in some cases be due to a similar genetically programmed response to adverse conditions.

## Results

### Cyanobacterial cell death accompanied by degradation of cyanobacterial DNA but persistence of cyanobacterial proteins

We subjected an alkaliphilic, mat-forming, cyanobacterial consortium to a 12-day dark and anoxic incubation, comparable to what it might experience upon sinking or sediment burial in its natural soda lake habitat. We collected samples from the solids (mat biomass) and supernatant (lysed and free-living) fractions every two days that were used for metagenomics and metaproteomics analysis. This data was then examined for signs of death-causing agents including viral attack, predation, and programmed cell death.

**Fig. 1:**
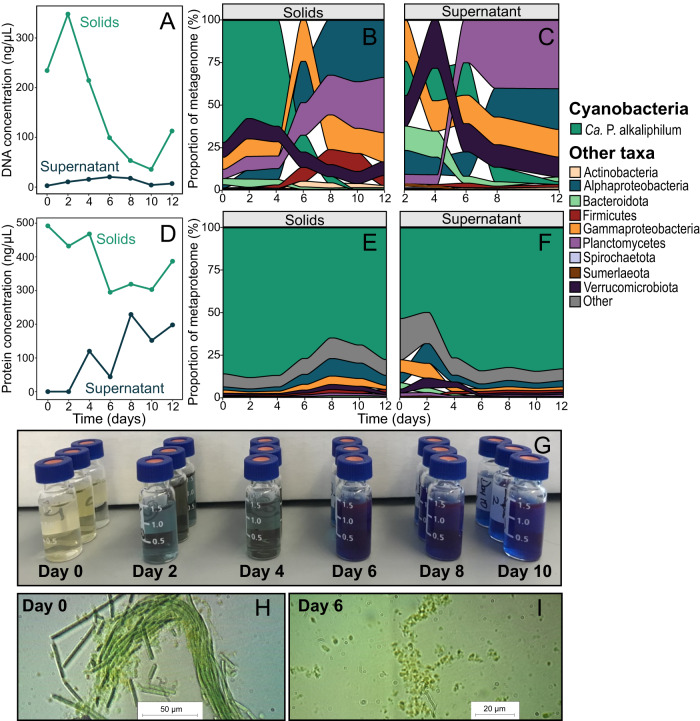
Observations during the 12-day dark and anoxic incubation. Concentration of DNA (**A**) and protein (**D**) in the solid and supernatant fractions. The microbial composition of the consortium is determined from the DNA in the solids (**B**), and supernatant (**C**) fractions, and from the protein content in the solids (**E**), and supernatant (**F**) fractions. Each time point from metagenome and metaproteome analysis represents a sacrificed independent experiment. Image showing the color of the supernatant fraction of each sample taken during the incubation (**G**). Brightfield microscopy images of the cyanobacterial consortium were taken on day 0 (**H**), and day 6 (**I**) of the incubation.

Initially, 72 % of the DNA extracted from the solid fraction originated from *Ca*. P alkaliphilum, but only 3.6 % of this DNA remained after 6-8 days in the dark. After 12 days, DNA from *Ca*. P alkaliphilum was barely detected (0.15%) (Fig. 1B). A similar pattern was observed in the supernatant fraction, as initially 20% of DNA could be attributed to *Ca*. P. alkaliphilum, which decreased to 1.6% by day 12 (Fig. 1C). In contrast, cyanobacterial proteins persisted throughout the incubation, always making up at least 65% of the protein composition in the solids fraction (Fig. 1E) and increasing to >80% of the supernatant fraction (Fig. 1F), suggesting a discrepancy in the way that the two biomolecules were degraded. Coinciding with the decrease of cyanobacterial DNA was an increase in concentrations of fermentation products such as acetate and propionate (Fig. S1) (Mann–Kendall Test: acetate: 0.714, *p* < 0.05; propionate: 0.905, *p* < 0.01). Because acetate mainly accumulated before cyanobacterial lysis, *Ca*. P. alkaliphilum itself was likely responsible for its production [23, 28]. Propionate increased later in the incubation and was likely produced by other bacteria fermenting compounds within the cyanobacterial lysate. By day 6, the supernatant was colored intensely blue (Fig. 1G) and contained a large amount of the internal antenna protein, phycocyanin, based on UV/Vis spectrometry (Fig. S2). Proteomics showed that phycocyanin made up 22%–32% of the protein in supernatant samples (Fig. S3). Microscopy showed that the cyanobacterial cells were lysing and breaking apart by the sixth day of incubation, explaining the presence of phycocyanin in the external medium (Fig. 1). Cell lysis of densely populated cyanobacterial blooms and the subsequent blue color change caused by the release of phycocyanin is a phenomenon that has been observed previously in freshwater lakes, but the mechanism of these lysis events remains unknown [29, 30].

### Minimal evidence for cell death due to predation or viral attack

**Fig. 2:**
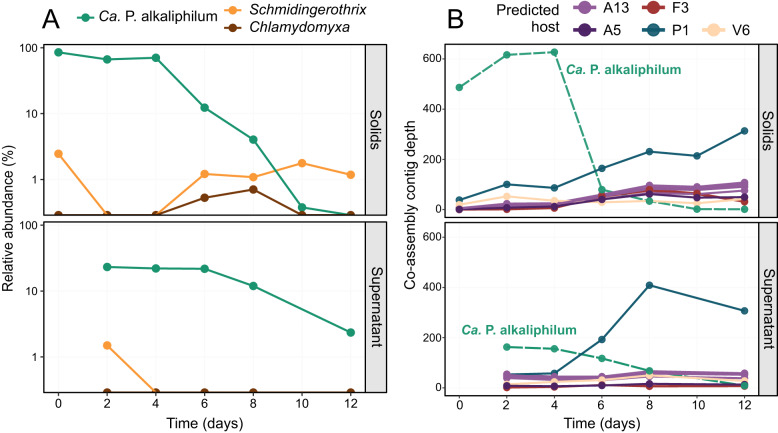
Dynamics of protist and viral sequences over the incubation. Relative abundance of *Ca*. P. alkaliphilum (green) and the two eukaryotic species identified (orange and brown), over the course of the incubation (**A**). Relative abundance based on rRNA genes retrieved from metagenomes using *PhyloFlash*. Y-axis is presented as a log-scale. Mann-Kendall Test: *Schmidingerothrix*: 0.1, *p* = 0.9; *Chlamydomyxa* = 0.2, *p* = 0.7; both Eukaryotes: 0, *p* = 1. Abundance of viral associated contigs over the incubation (**B**). The figure displays the top ten most abundant contigs with potential viral association. The top ten viral contigs had high confidence BLAST hits (>98% sequence identity) to contigs from MAGs, so the viral contigs are colored based on their potential bacterial host. The taxonomy of the predicted hosts are A13: Rhodobacteraceae, A5: *Pararhodobacter*, F3: *Alkalibacterium*, P1: UBA6054 (genus of *Planctomycetota*), V6: Verruco-01 (family of *Verrucomicrobiota*). The green dashed line shows the average contig depth for the *Ca*. P. alkaliphilum co-assembly MAG over the same samples. Each time point from the metagenome analysis represents a sacrificed independent experiment.

The possibility of predation by a larger eukaryotic cell or an antagonistic bacterial species was evaluated using the metagenomics data. Eukaryotic ciliate grazers affiliated with *Schmidingerothrix* were present in the consortium, however their abundance, calculated through copies of the rRNA gene in the metagenomes, was low (<2%) and did not increase over the course of the incubation (Fig. 2A) (Mann–Kendall Test: 0, *p* = 1), suggesting that eukaryotic predation was not directly responsible for the collapse of the cyanobacterial population.

Provisional genomes, or metagenome-assembled genomes (MAGs), were acquired for the main cyanobacterial species, *Ca*. P. alkaliphilum, in addition to 59 other species from eight bacterial phyla including *Proteobacteria*, *Bacteroidota*, *Firmicutes*, *Planctomycetota*, and *Verrucomicrobiota* (Fig. 1, Table S1). The observed bacterial dynamics, and the diverse and enhanced expression of carbohydrate-active transporters, like the TonB-dependent transporters (expressed by at least 24 different MAGs), are akin to the succession of bacterial populations after phytoplankton blooms in other aquatic systems [31]. However, although the relative abundance of heterotrophic DNA increased during the incubation (Mann-Kendall Test: 0.9, *p* < 0.01), the abundance of heterotrophic proteins did not increase significantly (Mann–Kendall Test: 0.4, *p* = 0.2) (Fig. 1) and no single species benefited in proportion to the magnitude of the cyanobacterial decline. Total DNA concentrations decreased (Fig. 1A) (Mann–Kendall Test: −0.9, *p* < 0.05), released cyanobacterial proteins persisted (Mann–Kendall Test: 0.1, *p* = 0.8), and fermentation (producing acetate) stabilized after the lysis of the cyanobacteria (Fig. S1) (Mann–Kendall Test; acetate on days >4: 0, *p* = 1). This might mean that the mainly aerobic consortium members did not have time to consume the released proteinaceous cyanobacterial lysate anaerobically. Taken together, our data did not point to a primary role of antagonistic bacteria in the cell lysis event.

Cyanophages, viruses that specifically target cyanobacteria, play an important role in the fate of cyanobacteria in natural environments, and consequently in global carbon and nutrient cycles [32, 33]. *Ca*. P. alkaliphilum contains seven CRISPR arrays, suggesting previous viral infections [34]. However, no viral contigs with regions of sequence identity to the *Ca*. P. alkaliphilum genome was identified in the assembly [35] (Fig. 2B). The most abundant contig of viral origin identified by *Virsorter* [36] was inferred to be a prophage of a *Planctomycetota*, and not associated with *Ca*. P. alkaliphilum. It also only reached 50% of the average cyanobacterial pre-collapse sequencing depth (Fig. 2B). In the event of a viral-mediated lysis, the depth of the associated viral contigs would be expected to increase at least 10-fold (burst size) in comparison to the depth of the cyanobacterial host contigs [37]. The comparatively low abundance of viral-associated contigs indicated that a mass viral-induced lysis of the cyanobacterial cells did not occur.

### Extensive changes in cyanobacterial proteome allocations pre and post lysis suggest genetic programming led to cell death

We explored if a genetically programmed signal was the most likely cause of cyanobacterial lysis. Total cyanobacterial protein abundance in the community remained relatively unchanged during the 12-day incubation (Mann–Kendall Test: −0.238, *p* = 0.5), and included detection of just over 2000 proteins, accounting for 52% of the predicted proteome of *Ca*. P. alkaliphilum (Table S2). Of the expressed proteins, 459 increased by at least two-fold between the beginning and the end of the dark incubation, while 1,039 proteins decreased expression by over 50%. In general, cyanobacteria do not extensively change their proteome composition in response to diel cycling [38, 39]. Thus, a greater than twofold change in approximately 75% of the expressed proteins suggests that *Ca*. P. alkaliphilum had mounted a stress response that was outside the normal range of proteomic circadian cycles.

**Fig. 3:**
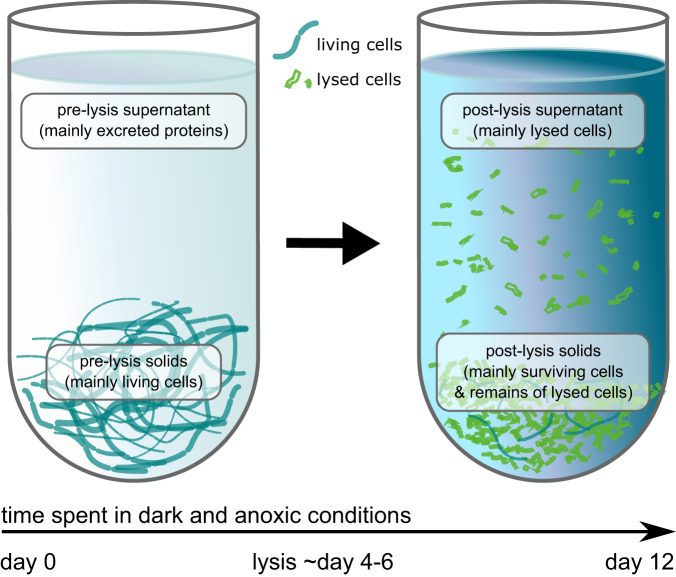
Depiction of sample fractions used for statistical analysis of protein dynamics. Schematic showing the expected cyanobacterial content in each fraction (grey boxes) tested in the point biserial correlation analysis.

To better understand protein dynamics, we conducted a point biserial correlation analysis. This analysis identified proteins that were statistically linked to different time periods and sample fractions during the incubations. The groups investigated in detail were the pre-lysis solids fraction (living stressed cells), the post-lysis solids fraction (surviving cells and insoluble proteins of lysed cells), and the post-lysis supernatant fraction (soluble proteins of lysed cells) (Fig. 3, Table S3). These groupings were chosen based on NMDS clustering of the cyanobacterial proteomics data (Fig. S4) and supernatant concentration of the intracellular pigment, phycocyanin (Fig. S2), which was used as a proxy for cell lysis. We also identified proteins associated with both the pre-lysis solids and the post-lysis supernatant (Fig. S5). This group likely contained typical intracellular proteins released into the media during lysis. In the following sections, we will provide more details about these findings.

There were 52 proteins significantly more abundant in the solids fraction post-lysis. These proteins could have been upregulated in response to stress, or they could represent the proteome of surviving persister cells. The proteins upregulated in the solids fraction post-lysis included subunits of ATP synthase, cytochrome b6f, Photosystem I (PSI), and Photosynthetic Complex I (formerly called NDH-I). The latter is a homolog of Respiratory Complex I and is known to form a supercomplex with PSI [40]. These changes are signs of a shift from linear electron flow to cyclic electron flow around PSI [41, 42] (Table S2). In cyclic electron flow, electrons are cycled around PSI via passage through cytochrome b6f complex, ferredoxin, and Photosynthetic Complex I [43–46]. This results in the formation of a proton gradient to generate ATP, but no formation of NADPH. In cyanobacteria, cyclic electron flow is favored when the cell is in an overly reduced state, and the ratio of ATP/NADPH is low [47]. This often occurs when the cell is in a stressed, energy-starved state due to conditions like darkness or drought [48, 49]. During the incubation, the relative expression of PSI increased nearly four-fold to 12.7% of the metaproteome by day 12. On this day, it was three-fold higher than the expression of Photosystem II (PSII), in contrast to the start of the experiment where PSI and II were expressed at an equal ratio. Proteins not required for cyclic electron flow, like ferredoxin-NADP+ reductase, and the oxygen-evolving complex of PSII decreased ten and two-fold, respectively, during the same period.

Previous studies have identified an increase in cyclic electron flow in response to dark and anoxic conditions and have hypothesized that it could be a defensive strategy in preparation for the return of daylight and resumption of photosynthesis [48], or alternatively as a mechanism to jumpstart metabolism through the rapid generation of ATP once light energy returns [49]. In our incubation, light did not return, and the mounted stress response was ultimately unsuccessful.

**Fig. 4:**
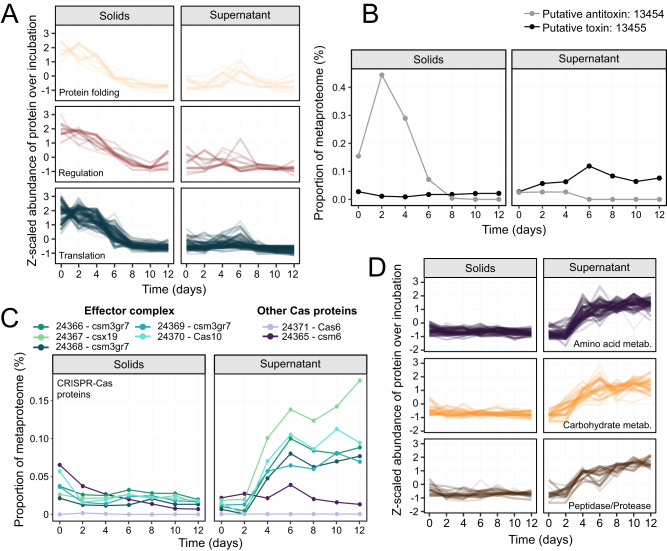
Dynamics of *Ca*. P. alkaliphilum proteins over the course of the incubation. Proteins from functional categories that were significantly more abundant in the solids fraction before cell lysis (**A**). Protein abundances were scaled across samples for visualization purposes. For a complete list of these proteins see Table S3. Metaproteome abundance of the prospective toxin-antitoxin protein pair over the incubation (**B**). The numbers indicate the protein accessions (Data S1). Abundance of proteins located in a CRISPR-Cas operon that were significantly enriched in the supernatant post-lysis (**C**). Numbers correspond to protein accession (Data S1). Proteins from functional categories that were significantly higher in abundance in the post-lysis supernatant fraction (**D**). Protein abundances were scaled across samples for visualization purposes. For a complete list of these proteins see Table S3.

Proteins that were statistically more abundant in the pre-lysis solids fraction, and downregulated before cell lysis, were largely involved in translation and transcription, and included ribosomal proteins, elongation factors, translation initiation factors, tRNA ligases, and RNA and DNA polymerases (Fig. 4A). Additionally, proteins expressed more in the pre-lysis solids fraction included protein chaperones (e.g., GroEL), and transcriptional regulators, most with poorly defined functions and targets (Fig. 4A). During the incubation, the abundance of these proteins decreased significantly, by as much as 45-fold, and there was no corresponding increase observed in the post-lysis supernatant fraction. This indicates that these proteins were actively degraded rather than being released into the surrounding media during lysis (Fig. S5).

Within the *Ca*. P. alkaliphilum genome, there are multiple toxin-antitoxin systems [23]. One particular putative antitoxin protein (accession: 13454) was observed to have a higher abundance in the pre-lysis solids fraction and was undetectable in the post-lysis incubation (Fig. 4B). This protein shares sequence similarity with the transcriptional regulator AbrB [50, 51] and the MazE antitoxin [52]. Both of these proteins are known to bind and inhibit the transcription of toxin genes [53, 54]. The specific toxin associated with this putative system remains unknown, but it is possible that the protein directly downstream (accession: 13455) could fulfill the role, as it possesses a ribonuclease domain and showed significantly higher abundance in post-lysis supernatant samples, increasing 4-fold in abundance from day 0 to peak lysis on day 6 (Fig. 4B). In MazEF toxin-antitoxin systems, the toxin MazF often functions as an endoribonuclease [52], exerting its toxicity by inhibiting protein synthesis [17, 55, 56]. MazF has also been implicated in the selection of cells that undergo cell death versus those that survive under stressful conditions [57]. This association is speculative because the study of toxin-antitoxin systems in prokaryotes is still relatively new and has primarily focused on model organisms [58], leading to a potential underestimation of the full diversity of these systems.

In conducting this differential abundance analysis, it was important to distinguish between intracellular proteins that are naturally released into the media during the lysis process and proteins that were statistically more abundant in the post-lysis supernatant fraction, indicating upregulation in lysing cells. We differentiated these two groups by distinguishing proteins that were enriched in both the solid pre-lysis and supernatant post-lysis fractions (typical intracellular proteins) from proteins that were enriched exclusively in the supernatant post-lysis fraction (potentially involved in lysis). There were 16 proteins that showed significant association with both the pre-lysis solids and post-lysis supernatant fractions. These proteins primarily included antenna-complex proteins such as phycobilisome linker proteins and allophycocyanin, as well as proteins involved in carbon metabolism like RuBisCO, phosphoglycerate kinase, and transketolase (Fig. S5).

A total of 142 proteins showed a significant increase in abundance exclusively in the post-lysis supernatant fraction. These proteins were likely upregulated in cyanobacterial cells shortly before their death and subsequently released into the surrounding media during lysis. As a result, it is plausible that these proteins played a role in causing cyanobacterial death. Several CRISPR-associated (Cas) proteins were significantly enriched in the post-lysis supernatant fraction. A set of five Cas proteins forming a Type III CRISPR-Cas operon (accessions: 24366-24370) were highly expressed in the supernatant post-lysis. Expression of these Cas proteins increased over 10-fold between pre- and post-lysis in the supernatant (Fig. 4C). Because we were unable to find evidence for viral lysis of *Ca*. P alkaliphilum (Fig. 2B), it seems possible that this Cas response was independent of viral or immune activity and may instead be associated with the cyanobacterial response to stress. In support of this theory, previous research has suggested that ancestral CRISPR-Cas effectors were stress response systems that triggered programmed cell death after activation by a signaling molecule [59, 60]. CRISPR Type III effector complexes consist of a Cas10 protein and other subunit proteins Csm3 (Cas7), and Csx19, acting as a multi-subunit nuclease [61, 62]. The nuclease activity of the Type III effector protein complex has previously been linked to cell death and dormancy [63]. Other components of the Type III system include Cas1 and Cas2 (accessions: 24372-3), involved in acquiring CRISPR spacers from foreign DNA, and Cas6 (accession: 24371), an endonuclease involved in splicing CRISPR RNA to guide effector complexes. Over the course of the incubation, neither Cas1 nor Cas2 was expressed, and Cas6 was very low abundance (<0.002%), suggesting that only the Cas effector complex was active and not the Cas components necessary for spacer acquisition and guide RNA generation. The protein Csm6 (accession: 24365) generally followed similar dynamics to the effector complex Cas proteins, with a supernatant peak on day 6, but was statistically higher in the solid pre-lysis fraction (Fig. 4C). In canonical Type III CRISPR-Cas systems, Csm6 is the enzyme responsible for initiating cell death by non-specific degradation of host and phage transcripts and is activated by cyclic-oligoadenylate second messengers produced by the effector complex [64]. Csm6 is known to be allosterically regulated by the second messenger, which is also its substrate, and thus could have activity levels that vary from expression levels [64].

Proteins associated with the supernatant post-lysis fraction also included several peptidases and proteases, as well as over 30 proteins involved in amino acid metabolism (Fig. 4D). This indicates active proteome remodeling or degradation. Additionally, several proteins involved in carbohydrate degradation and synthesis, as well as electron and iron carriers such as ferredoxin and bacterioferritin were enriched in the supernatant post-lysis.

Could energy depletion in the dark have triggered the observed potential programmed cell death response? If cell lysis was triggered by energy depletion, increasing salinity would result in a need for higher cellular maintenance energy to cope with elevated osmotic pressure and would consequently deplete energy reserves sooner. This hypothesis was tested by performing independent dark and anoxic incubations of the cyanobacterial consortium at higher (1 M Na^+^) and lower (0.25 M Na^+^) salinity.

The dark and anoxic incubation at 0.25 M Na^+^, resulted in a cyanobacterial lysis event that occurred later and was less complete, characterized by a much lower concentration of released phycocyanin (1.5 mg/mL), compared to the original incubation at 0.5 M Na^+^ (6.6 mg/mL) (Fig. S6). In the incubation with 1 M Na^+^, cell lysis occurred sooner, by day 5 (Fig. S6), and the final concentration of phycocyanin was high (7.2 mg/mL). These results support the hypothesis that cyanobacterial cell lysis in these dark and anoxic incubations is initiated by depleted energy reserves. Cells in an environment of higher salinity require more energy to maintain osmotic equilibrium, and thus deplete energy reserves faster.

### Rapid disappearance of cyanobacterial DNA in source soda lake sediment

**Fig. 5:**
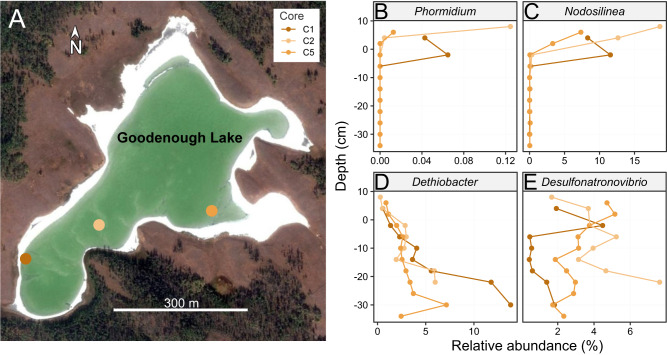
Microbial abundance in soda lake sediment cores. **A** Map of sampling locations within Goodenough Lake, British Columbia, Canada. Distribution of genera from 16S rRNA gene abundance: (**B**) Cyanobacteria *Phormidium* (*Ca*. P. alkaliphilum), (**C**) Cyanobacteria *Nodosilinea*, (**D**) Sulfidogenic thiosulfate and elemental sulfate reducing bacteria *Dethiobacter*, (**E**) Sulfidogenic, sulfate, sulfite and thiosulfate reducing bacteria *Desulfonatronovibrio*. Positive depth values in **B**–**E** represent the benthic cyanobacterial mat and negative centimeters represent distance below the sediment surface.

Evidence for similar cyanobacterial lysis occurring in situ was investigated using 30 cm cores of cyanobacterial mats and underlying sediment from the culture source, alkaline Lake Goodenough (Canada) (Fig. 5A). 16S rRNA gene amplicon sequencing of sectioned cores showed a high abundance of cyanobacteria at the top of the mat (Fig. 5B, C). The abundance of cyanobacteria like *Phormidium* and *Nodosilinea* decreased rapidly and became essentially negligible two cm below the sediment surface. Rapid turnover of cyanobacterial biomass could explain the previously observed steep sulfide gradients [25]. Sulfide likely builds up below the mats after the depletion of oxygen because sulfur-reducing bacteria oxidize fatty acids, hydrogen, and other cyanobacterial degradation products and reduce sulfate and other sulfur-compounds to sulfide. Amplicon sequencing showed ecological success at depth of thiosulfate and elemental sulfur-reducing *Dethiobacter* [65, 66], and the sulfate, thiosulfate, and sulfite reducing *Desulfonatronovibrio* [67] (Fig. 5D, E).

## Discussion

Despite the importance associated with biomass turnover in cyanobacterial systems, there is a general lack of understanding of what causes the collapse of dense cyanobacterial mats and blooms, and what occurs at the molecular level in these cells before death. Here we used metagenomics and metaproteomics to gain insights into the molecular state of the cyanobacterial cells prior to and post lysis. Based on these data, the most probable conclusion is that the mass cell lysis event was caused by programmed cell death in response to severe energy limitation and osmotic stress.

Proteogenomics showed that the cells altered their proteome to combat energy stress brought about by darkness, by the rearrangement of protein complexes in the thylakoid membrane to favor cyclic electron flow. Fermentation of available endogenous carbohydrates, like glycogen, cyanophycin, or the osmolytes sucrose, glucosyl glycerol, and trehalose, would have initially provided energy for sustaining cellular integrity and resulted in the observed increase in acetate [68, 69] (Fig. S1). After four days, proteins involved in transcription and translation were severely diminished in the solids fraction, a signal of decreased metabolism and arrested growth. With continued darkness, the supply of endogenous carbohydrates and osmolytes was likely depleted [70]. The ensuing starvation and osmotic stress may have triggered a programmed cell death response, possibly through toxin-antitoxin systems, proteases, and/or the expression of Type III CRISPR-Cas proteins in an upregulated operon, resulting in lysis of the cyanobacterial cells.

In unicellular organisms, programmed cell death presents an intriguing evolutionary puzzle, as it raises questions about the obvious fitness cost to the organism when it ultimately perishes, eliminating any further opportunity for its own reproduction. Recent work has argued that prokaryotic programmed cell death can be viewed in light of the Black Queen Hypothesis, where public goods arising from a leaky and costly function performed by a few, are exploited by other members of the community [71]. Programmed cell death is both leaky, allowing members of the community to use compounds from the expelled lysate, and costly to the perpetrator, ending in the demise of the participating organism. But, due to the potential overall benefit to the broader community, it may provide an inclusive fitness advantage to close relatives. Several experimental studies have provided support for this theory. Previous work on *E. coli* has shown that groups with programmed cell death persevered when challenged with an obligately lytic prophage, and groups without programmed cell death went extinct [72]. In the unicellular alga, *Dunalleila salina*, programmed cell death was used to complement the nutritional needs of other cells of the same species that didn’t possess this trait [73]. In that study, heterotrophs also benefited from the released lysate, re-mineralizing the dissolved organic matter which further promoted algae growth [73]. In work focusing on the unicellular alga, *Chlamydomonas reinhardtii*, programmed cell death was found to benefit members of the same species, but inhibited members of other species [74]. In the natural habitat of *Ca*. P. alkaliphilum, a complex microbial mat containing multiple cyanobacterial species [22], this particular outcome of programmed cell death could infer a fitness advantage by reducing competition with other cyanobacteria. The allelopathic effects of programmed cell death in this context would be a very interesting subject for further study.

Evidence for programmed cell death has been previously identified in cyanobacterial species like *Trichodesmium* [12, 75], and *Microcystis* [11, 76]. Furthermore, a similar lysis phenomenon was observed in dark and anoxic incubations of a thermophilic cyanobacterium, *Oscillatoria terebriformis*, isolated from hot spring microbial mats [77]. In that experiment, cell survival could be prolonged by the addition of an exogenous carbohydrate source (fructose), and/or a reductant (e.g., sodium thioglycolate). This might be another example where dwindling energy stores cause the lysis of a cyanobacterium under dark and challenging conditions. The addition of fructose sustained cell survival by providing another substrate for cyanobacterial fermentation. Whereas the addition of reducing agents could have quenched reactive oxygen species (ROS) produced under stress. A link between ROS and programmed cell death in both eukaryotic and prokaryotic cells has long been known [14, 78, 79], and the production of ROS has previously been associated with the activation of toxin-antitoxin systems upon stress [80, 81].

Further study of this cell death process in cyanobacteria may lead to new avenues to control harmful algal blooms. Detection of free phycocyanin in lakes after blooms [82] already indicates that the same process could be relevant and, with follow-up research, manipulated. Furthermore, cyanobacterial cellular lysis due to dark and anoxic incubation provided a way to access the internal pigment phycocyanin without costly and energetically intensive mechanical disruption [83]. Adapting this process to an industrial setting could reduce the costs and energy associated with the production of phycocyanin and other internal compounds.

This study provides important insights into mechanisms of mass cell death in cyanobacterial mats and blooms. From this data, we show that cellular machinery related to protein synthesis is specifically degraded during a prolonged dark period and hypothesize that there are previously unidentified roles for toxin-antitoxin systems and CRISPR-Cas proteins in cyanobacterial cell death. Additionally, this process could be a promising target for new antibiotics or algicidal compounds and harnessed as a commercially sustainable way to produce bioproducts.

## Materials and methods

### Experimental setup and sampling

An alkaliphilic cyanobacterial consortium enrichment culture containing a single, abundant, cyanobacteria species, *Candidatus* Phormidium alkaliphilum (renamed to “*Sodalinema alkaliphilum*” in GTDB version 214) [21, 23, 24], was used as inoculum for the dark incubation. This consortium was originally sourced from microbial mats of alkaline soda lakes in the Cariboo Plateau region of Canada [22], where *Ca*. P. alkaliphilum accounted for up to 2.5% of the DNA, and 7.5% of the protein (MAG C5 in that study). The cyanobacterial consortium was grown in continuous light (200 µmol photons/(m^2^·s)) in 10 L stirred glass vessels. The growth medium was previously described [84] and contained 0.5 M sodium (bi)carbonate alkalinity, at an initial pH of 10.3. After six days of photoautotrophic growth, the culture was centrifuged for 30 min at 4500 rpm to concentrate the biomass (Allegra X-22R, Beckman Coulter, USA). The wet biomass was then divided into 20 mL serum bottles sealed with butyl-rubber septa. Two grams of wet biomass were added to each serum bottle. The bottles were purged with N_2_ gas to create an anoxic headspace and then placed at room temperature (21 °C) in the dark. At 0, 2, 4, 6, 8, 10, and 12 days after the start of the incubation, two sacrificial samples were taken. To each sample, 5 mL of pH 7, phosphate-buffered saline solution was added and then the sample was centrifuged for 10 min at 4500 rpm, to separate biomass and supernatant.

For biomass pellets, the ash-free dry weight of each sample was measured using NREL laboratory analytical procedures protocol [85]. For the supernatant, the concentration of the organic acids acetate, formate, propionate, butyrate, succinate, and lactate were measured using an UltiMate 3000 HPLC system (ThermoFisher Scientific, USA) equipped with an Aminex HPX-87H column and a UV detector, as previously described [86]. The phycocyanin and total protein concentration in the supernatants were measured as absorption at 620 nm and 280 nm respectively [87] using an Evolution 260 Bio UV-Visible Spectrophotometer (ThermoFisher Scientific, USA), with a standard curve prepared from laboratory-grade phycocyanin (Sigma-Aldrich, USA). Bright-field microscope images were taken using a Zeiss Axio Imager A2 Microscope (Carl Zeiss AG, Germany).

### DNA extraction

DNA was extracted directly from soda lake sediment and incubated solid samples using the Fast DNA Extraction Kit for Soil (MP Biomedicals, USA). For the incubation of supernatant samples, 250 µL of the sample was used for each extraction. The extraction protocol of the manufacturer was followed, but additional purification steps were performed with 5.5 M guanidine thiocyanate [21]. For supernatant samples, the elution buffer was heated to 50 °C prior to the elution step to increase yield. Still, supernatant samples from Day 0 and Day 10 did not yield enough DNA for metagenome analysis.

### 16S rRNA gene PCR and sequencing

Amplicon sequencing and library preparation of the DNA sediment samples were performed as previously described [21] using primer sets 926wF (5′-AAACTYAAAKGAATTGRCGG3′) and 1392 R (5′-ACGGGCGGTGTGTRC3′) targeting bacteria [88, 89]. Prepared libraries were sequenced on the MiSeq Personal Sequencer (Illumina, USA) using the 2 × 300 bp MiSeq Reagent Kit v3. Amplicon sequencing results were processed using MetaAmp Version 3.0 [90], and the Silva database version 138 [91]. Paired-end reads were merged if they had less than eight mismatches in the overlap region and overlap of >100 bp [21, 92]. The merged reads were further filtered by removing reads that were missing the forward or reverse primer and had more than one mismatch in the primer region. All reads were trimmed to a final of 350 bp and clustered into operational taxonomic units (OTUs) of >97% sequence identity [21, 92]. One OTU was classified as “*Geitlerinema”* by Silva and shared 100% sequence identity with the 16S rRNA gene from *Ca*. P. alkaliphilum. For consistency, this OTU was relabelled “*Phormidium”* in Fig. 5B.

### Library preparation and metagenome sequencing

All biomass samples, and supernatant samples from days 2, 4, 6, 8, and 12 were prepared for metagenomic sequencing as previously described [22]. Briefly, DNA was sheared to fragments of ~300 bp and libraries were created using the NEBNext Ultra DNA Library Prep Kit (New England Biolabs, Ipswich, MA). A NextSeq 500 sequencer (Illumina, San Diego, CA) was used for sequencing using a 300 cycle (2x150bp) high-output sequencing kit at the Center for Health Genomics and Informatics in the Cumming School of Medicine, University of Calgary, Canada.

### Metagenome assembly, binning, and analysis

Raw, paired-end Illumina reads were filtered for quality using BBDuk (https://jgi.doe.gov/data-and-tools/bbtools/). Quality control consisted of trimming reads to 150 bp, trimming off adapter sequences, filtering out contaminants, such as the PhiX adapter, and clipping off low-quality ends, all as previously described [22]. Paired-end reads from each sample were then merged with BBMerge [93]. Separate assemblies of the reads from each sample were performed using metaSPAdes version 3.12.0 with default parameters [94]. To increase binning success, one large co-assembly using the unmerged reads from all samples was conducted using MegaHit v1.2.2 [95]. Only contigs greater than 500 bp in length were processed further. The MetaErg pipeline [96] was used for the prediction and annotation of genetic elements on each assembled contig.

Binning of assembled reads into metagenome-assembled-genomes (MAGs) was completed using MetaBat2 version 2.12.1 [97]. The binning step was performed on each sample’s assembly separately as well as the co-assembly. To generate sequencing depth data for binning, quality-controlled reads of each sample were mapped to the assembly of each sample using BBMap v38.84 (https://sourceforge.net/projects/bbmap/). Mapping results were summarized using the script, “*jgi_summarize_bam_contig_depths*”, part of the MetaBat package [98]. After binning, the program dRep [99] in conjunction with CheckM v1.0.11 [100] was used to determine the best (highest estimated completeness, and lowest estimated contamination) MAGs associated with each population. In total, 60 MAGs (>80% completeness, and <5% contamination) were identified for further processing and analysis. The program, gtdbtk v0.3.2 was used for the taxonomic assignment of each MAG [101].

The relative abundance of individual MAGs in each metagenome was calculated by mapping quality-controlled raw reads from each sample onto the contigs of each MAG as well as the dereplicated contigs that remained unbinned. Again, BBMap (minid = 0.98) was used for this. Unbinned contigs were dereplicated using cd-hit-2d [102]. In this step, all contigs sharing > 90% sequence identity with a binned contig were eliminated. The number of reads that mapped to each contig was counted, and then the total counts for each contig of each MAG were totaled. To determine relative abundance, counts were normalized to MAG genome size and the number of mapped reads per sample. For the unbinned contigs, reads were normalized to the number of base pairs in all dereplicated unbinned contigs.

The program phyloFlash v3.3 (*Emirge* assembly) was used to obtain full-length 16S and 18S ribosomal RNA (rRNA) gene sequences and their sequencing depth from the metagenomes [103]. The sequencing depths of rRNA sequences were used primarily to determine the population dynamics of species that did not assemble or form MAGs well, mainly eukaryotic protists.

Mann-Kendall tests, using the command *MannKendall* from the R package *Kendall* [104], were performed on various measures of the time series data (e.g., abundance of Eukaryotes and heterotrophs, organic acid concentrations, DNA, and protein concentrations) to determine the presence of a statistically significant trend over time. If the reported *p* value was <0.05, then the statistic value reported, *tau*, determined the direction of the statistically significant trend, with *tau* > 0 signifying positive trends over time, and *tau* < 0 signifying negative trends over time.

### Analysis of viral contigs

Contigs potentially associated with lytic and temperate viruses were identified from the metagenome co-assembly using VirSorter v1.0.6 [36]. BLASTn was then used to match the DNA sequence of viral contigs to the 60 MAGs. CRISPR arrays in the cyanobacterial MAG were identified from the MetaErg output [96], with the program MinCED (github.com/ctSkennerton/minced).

### Protein extraction and LC-MS/MS mass spectrometry

Protein was extracted from biomass and supernatant samples as previously described [105], using the filter aided sample preparation (FASP) protocol [106]. To lyse cells, samples were added to lysing matrix E bead tubes (MP Biomedicals, USA) with SDT-lysis buffer (0.1 M DTT) in a 1:10 sample-to-buffer ratio. The tubes were then subjected to bead-beating in an OMNI Bead Ruptor (Omni International, USA) 24 for 45 s at 6 m s^−1^. For supernatant samples, 500 µL of supernatant was used for lysis. Supernatant samples from days 0 and 2 had low yields so lysate was concentrated prior to protein extraction.

Peptides were separated by an UltiMate 3000 RSLC nano Liquid Chromatograph (Thermo Fisher Scientific, USA), using a 75 cm × 75 µm analytical column and analyzed in a Qexactive Plus hybrid quadrupole-Orbitrap mass spectrometer (Thermo Fisher Scientific, USA) as previously described [107]. A total of 2000 ng of peptide was loaded, and each sample was run for 4 h.

### Metaproteomics data analysis

The database used for protein identification was manually created using the predicted and annotated proteins from the binned and unbinned metagenomic sequences. Cd-hit was used to remove redundant sequences from the database using an identity threshold of 95% [102], giving preference to sequences that came from MAGs. Sequences of common contaminating proteins were added to the final database (http://www.thegpm.org/crap/). The final database contained 454,164 proteins and preserved the assignment of predicted proteins to their respective MAGs from the metagenomes. This enabled the calculation of proteome contribution for each MAG. For protein identification MS/MS spectra were searched against the database using the Sequest HT node in Proteome Discoverer version 2.2.0.388 (Thermo Fisher Scientific, USA) as described previously [108]. Only proteins with one unique peptide, and with a protein false discovery rate (FDR) confidence of at least a level of “medium”, were kept for further analysis.

Relative protein abundances were estimated using the normalized spectral abundance factor (NSAF) [109]. MAG abundance in the metaproteome was estimated by adding the NSAF abundance of all proteins belonging to that MAG. In total, 3,286,730 MS/MS spectra were obtained, yielding 632,137 peptide spectral matches (PSMs), which corresponded to 10,408 expressed proteins after quality control.

To further investigate the protein expression dynamics during the dark and anoxic incubation, the *Ca*. P. alkaliphilum proteomics data was divided into pre and post lysis groupings. The supernatant pre lysis grouping included days 0 and 2, and the supernatant post lysis grouping included days 4–12. The solids pre lysis grouping included days 0, 2, and 4, and the solids post lysis grouping included days 6–12. These groupings were chosen based on NMDS clustering (Fig. S4) of the cyanobacterial proteomics data and the phycocyanin concentration in the supernatant (Fig. S2), which was used as a proxy for cell lysis. NMDS was performed using the metaMDS function from the R package *vegan* [110] based on Bray-Curtis dissimilarity of proteins with greater than 50 PSMs. Proteins that were statistically differentially abundant between these groupings from the *Ca*. P. alkaliphilum MAG was identified using point biserial correlation implemented with the *multipatt* function from the *indicspecies* package in R [111] (Table S3). The *multipatt* function was run with the “r.g” function and 9999 permutations. Only proteins that had at least 50 PSMs in total were included in the analysis, and a *p* value of 0.05 was needed to be considered significant. The functional annotations of statistically significant proteins were further validated using BLASTP searches against the NCBI nr database.

### Sediment sample collection and preparation

Duplicate sediment cores were collected in April 2019 from Goodenough Lake (51.330°N, 121.64°W). The sediment cores were taken from 3 different locations within each lake (Fig. 1) using a 1.5-m single-drive Griffith corer from LacCore: National Lacustrine Core Facility (University of Minnesota). The sediment cores ranged in length from 25–50 cm. To reduce the mixing of water and upper sediment layers in the cores, Zorbitrol was used as a gelling agent to stabilize the sediment-water interface during transport. Cores were then stored upright at −20 °C.

Cores were removed from the −20 °C freezer and defrosted at room temperature (22 °C) for 2 h. Cores were then horizontally sliced into 2 cm disks using a Dremel Multi-Max MM50 oscillating saw (Dremel, USA) at the lowest speed, used to reduce blade contact with the sediment. The blade was sterilized with 70% ethanol before each core section was sampled. To avoid the potential risk of contamination from the core liner or during sectioning, sediment in contact with the core liner was removed, and the inner core was transferred to a 50 mL tube, sealed, and stored at −20 °C. The sediment from each disk was subsampled for DNA extraction and stored at −80 °C.

### Sodium, biomass concentration, and Spirulina experiments

The dark and anoxic incubation of the cyanobacterial consortium was repeated using dewatered biomass with different concentrations of sodium. Initially, biomass obtained after growth was first dewatered and then gently washed with deionized water to remove the salts. This step was repeated five times to ensure that all the salts were removed. The washed biomass was then separated into three aliquots. Each aliquot was washed with sodium carbonate solution with varied concentrations (0.25 M, 0.5 M, and 1 M). Then, approximately 2 g of wet paste from each aliquot was placed in sterile serum bottles. The headspace in the serum bottles was vacuumed and filled with N_2_ gas up to atmospheric pressure to create anoxic conditions. These serum bottles were then incubated in the dark at room temperature for eight days. Every day, two bottles were removed from the incubation and analysed for phycocyanin as described above. Electron microscopy was conducted as described previously [112] without performing ethanol washes and without using gold-sputtered filters.

### Supplementary information

Supplementary Material

Supplementary Table 1

Supplementary Table 2

Supplementary Table 3

Supplementary Data 1

## Data Availability

All data needed to evaluate the conclusions in the paper are present in the paper and/or the Supplementary Materials. Metaproteomes were deposited to the PRIDE database under accession PXD023504. Metagenomes can be found in BioSamples SAMN17264972-SAMN17264985 (BioProject: PRJNA377096). MAGs from the study were deposited into the NCBI Whole Genome Shotgun submission database under BioSamples SAMN17266165-SAMN17266224.

## References

[1] Huisman J, Codd GA, Paerl HW, Ibelings BW, Verspagen JMH, Visser PM (2018). Cyanobacterial blooms. Nat Rev Microbiol.

[2] Hou X, Feng L, Dai Y, Hu C, Gibson L, Tang J (2022). Global mapping reveals increase in lacustrine algal blooms over the past decade. Nat Geosci.

[3] Carmichael WW (2001). Health effects of toxin-producing cyanobacteria: “The CyanoHABs. ” Hum Ecol Risk Assess.

[4] Davies FK, Fricker AD, Robins MM, Dempster TA, McGowen J, Charania M (2021). Microbiota associated with the large-scale outdoor cultivation of the cyanobacterium *Synechococcus* sp. PCC 7002. Algal Res.

[5] Paerl HW, Otten TG (2013). Harmful cyanobacterial blooms: causes, consequences, and controls. Micro Ecol.

[6] Franklin DJ (2021). Examining the evidence for regulated and programmed cell death in cyanobacteria. How significant are different forms of cell death in cyanobacteria population dynamics?. Front Microbiol.

[7] Sigee DC, Selwyn A, Gallois P, Dean AP (2007). Patterns of cell death in freshwater colonial cyanobacteria during the late summer bloom. Phycologia.

[8] Bidle KD (2016). Programmed cell death in unicellular phytoplankton. Curr Biol.

[9] Trocshl C, Fritz I, Sodnikar K, Drosg B (2017). Contaminations in mass cultivation of cyanobacteria: highly resilient *Colpoda steinii* leads to rapid crash of *Synechocystis* sp. cultures and is inhibited by partially anoxic conditions. Algal Res.

[10] Van Wichelen J, Vanormelingen P, Codd GA, Vyverman W (2016). The common bloom-forming cyanobacterium *Microcystis* is prone to a wide array of microbial antagonists. Harmful Algae.

[11] Hu C, Rzymski P (2019). Programmed cell death-like and accompanying release of microcystin in freshwater bloom-forming cyanobacterium *Microcystis*: from identification to ecological relevance. Toxins.

[12] Berman-Frank I, Bidle KD, Haramaty L, Falkowski PG (2004). The demise of the marine cyanobacterium, *Trichodesmium* spp., via an autocatalyzed cell death pathway. Limnol Oceanogr.

[13] Bayles KW (2014). Bacterial programmed cell death: making sense of a paradox. Nat Rev Microbiol.

[14] Hochman A (1997). Programmed cell death in prokaryotes. Crit Rev Microbiol.

[15] Allocati N, Masulli M, Di Ilio C, De Laurenzi V (2015). Die for the community: an overview of programmed cell death in bacteria. Cell Death Dis.

[16] Bhattacharjee S, Mishra AK (2020). The tale of caspase homologues and their evolutionary outlook: deciphering programmed cell death in cyanobacteria. J Exp Bot.

[17] Harms A, Brodersen DE, Mitarai N, Gerdes K (2018). Toxins, targets, and triggers: an overview of toxin-antitoxin biology. Mol Cell.

[18] Koonin EV, Zhang F (2017). Coupling immunity and programmed cell suicide in prokaryotes: life-or-death choices. Bioessays.

[19] Koonin EV, Makarova KS (2022). Evolutionary plasticity and functional versatility of CRISPR systems. PLoS Biol.

[20] Steens JA, Salazar CRP, Staals RHJ (2022). The diverse arsenal of type III CRISPR-Cas-associated CARF and SAVED effectors. Biochem Soc Trans.

[21] Sharp CE, Urschel S, Dong X, Brady AL, Slater GF, Strous M (2017). Robust, high-productivity phototrophic carbon capture at high pH and alkalinity using natural microbial communities. Biotechnol Biofuels.

[22] Zorz JK, Sharp CE, Kleiner M, Gordon PMK, Pon RT, Dong X (2019). A shared core microbiome in soda lakes separated by large distances. Nat Comm.

[23] Ataeian M, Vadlamani A, Haines M, Mosier D, Dong X, Kleiner M (2021). Proteome and strain analysis of cyanobacterium *Candidatus* “Phormidium alkaliphilum” reveals traits for success in biotechnology. iScience.

[24] Ataeian M, Liu Y, Kouris A, Hawley AK, Strous M (2022). Ecological interactions of cyanobacteria and heterotrophs enhances the robustness of cyanobacterial consortium for carbon sequestration. Front Microbiol.

[25] Brady AL, Druschel G, Leoni L, Lim DSS, Slater GF (2013). Isotopic biosignatures in carbonate-rich, cyanobacteria-dominated microbial mats of the Cariboo Plateau, B.C. Geobiology.

[26] Stal LJ (1995). Physiological ecology of cyanobacteria in microbial mats and other communities. N. Phytol.

[27] Jorgensen BB, Revsbech NP, Blackburn TH, Cohen Y (1979). Diurnal cycle of oxygen and sulfide microgradients and microbial photosynthesis in a cyanobacterial mat sediment. Appl Environ Microbiol.

[28] Demirkaya C, Vadlamani A, Tervahauta T, Strous M, De la Hoz Siegler H (2023). Autofermentation of alkaline cyanobacterial biomass to enable biorefinery approach. Biotechnol Biofuels.

[29] Fallon RD, Brock TD (1979). Lytic organisms and photooxidative effects: influence on blue-green algae (cyanobacteria) in Lake Mendota, Wisconsin. Appl Environ Microbiol.

[30] Arii S, Tsuji K, Tomita K, Hasegawa M, Bober B, Harada K (2015). Cyanobacterial blue color formation during lysis under natural conditions. Appl Environ Microbiol.

[31] Teeling H, Fuchs BM, Becher D, Klockow C, Gardebrecht A, Bennke CM (2012). Substrate-controlled succession of marine bacterioplankton populations induced by a phytoplankton bloom. Science.

[32] Roux S, Brum JR, Dutilh BE, Sunagawa S, Duhaime MB, Loy A (2016). Ecogenomics and potential biogeochemical impacts of globally abundant ocean viruses. Nature.

[33] Puxty RJ, Evans DJ, Millard AD, Scanlan DJ (2018). Energy limitation of cyanophage development: implications for marine carbon cycling. ISME J.

[34] Khot V, Strous M, Dong X, Kiesser AK. Viral diversity and dynamics, and CRISPR-Cas mediated immunity in a robust alkaliphilic cyanobacterial consortium. bioRxiv. 2023. 10.1101/2023.03.03.531066. 10.1128/spectrum.02217-23 PMC10715143 37819096

[35] Edwards RA, McNair K, Faust K, Raes J, Dutilh BE (2016). Computational approaches to predict bacteriophage-host relationships. FEMS Microbiol Rev.

[36] Roux S, Enault F, Hurwitz BL, Sullivan MB (2015). VirSorter: mining viral signal from microbial genomic data. PeerJ.

[37] Weinbauer MG, Suttle CA (1996). Potential significance of lysogeny to bacteriophage production and bacterial mortality in coastal waters of the Gulf of Mexico. Appl Environ Microbiol.

[38] Welkie D, Zhang X, Markillie ML, Taylor R, Orr G, Jacobs J (2014). Transcriptomic and proteomic dynamics in the metabolism of a diazotrophic cyanobacterium, *Cyanothece* sp. PCC 7822 during a diurnal light-dark cycle. BMC Genom.

[39] Welkie DG, Rubin BE, Diamond S, Hood RD, Savage DF, Golden SS (2019). A hard day’s night: cyanobacteria in diel cycles. Trends Microbiol.

[40] Gao F, Zhao J, Chen L, Battchikova N, Ran Z, Aro E-M (2016). The NDH-1L-PSI supercomplex is important for efficient cyclic electron transport in cyanobacteria. Plant Phys.

[41] Hibino T, Lee BH, Rai AK, Ishikawa H, Kojima H, Tawada M (1996). Salt enhances photosystem I content and cyclic electron flow via NAD(P)H dehydrogenase in the halotolerant cyanobacterium *Aphanothece halophytica*. Aust J Plant Physiol.

[42] Zorz J, Allanach JR, Murphy CD, Roodvoets MS, Campbell DA, Cockshutt AM (2015). The RUBISCO to photosystem II ratio limits the maximum photosynthetic rate in picocyanobacteria. Life.

[43] Yamori W, Shikanani T, Makino A (2015). Photosystem I cyclic electron flow via chloroplast NADH dehydrogenase-like complex performs a physiological role for photosynthesis at low light. Sci Rep.

[44] Zhang C, Shuai J, Ran Z, Zhao J, Wu Z, Liao R (2020). Structural insights into NDH-1 mediated cyclic electron transfer. Nat Commun.

[45] Schuller JM, Birrell JA, Konuma T, Wulfhorst H, Cox N, Schuller SK (2018). Structural adaptations of photosynthetic complex I enable ferredoxin-dependent electron transfer. Science.

[46] Richardson KH, Wright JJ, Simenas M, Thiemann J, Esteves AM, McGuire G (2021). Functional basis of electron transport within photosynthetic complex I. Nat Commun.

[47] Takahashi H, Clowez S, Wollman F-A, Vallon O, Rappaport F (2013). Cyclic electron flow is redox-controlled but independent of state transition. Nat Commun.

[48] Golding AJ, Finazzi G, Johnson GN (2004). Reduction of the thylakoid electron transport chain by stromal reductants - evidence for activation of cyclic electron transport upon dark adaptation or under drought. Planta.

[49] Joliot P, Joliot A (2002). Cyclic electron transfer in plant leaf. Proc Natl Acad Sci.

[50] Bobay BG, Andreeva A, Mueller GA, Cavanagh J, Murzin AG (2005). Revised structure of the AbrB N-terminal domain unifies a diverse superfamily of putative DNA-binding proteins. FEBS Lett.

[51] Eraso JM, Markillie LM, Mitschell HD, Taylor RC, Orr G, Margolin W (2014). The highly conserved MraZ protein is a transcriptional regulator in *Escherichia coli*. J Bacteriol.

[52] Ning D, Jiang Y, Liu Z, Xu Q (2013). Characterization of a chromosomal type II toxin-antitoxin system *mazEaFa* in the cyanobacterium *Anabaena* sp. PCC 7120. PloS One.

[53] Zorzini V, Buts L, Schrank E, Sterckx YGJ, Respondek M, Engelberg-Kulka H (2015). *Escherichia coli* antitoxin MazE as transcription factor: insights into MazE-DNA binding. Nucleic Acids Res.

[54] Saile E, Koehler TM (2002). Control of anthrax toxin gene expression by the transition state regulator *abrB*. J Bacteriol.

[55] De Bruyn P, Girardin Y, Loris R (2021). Prokaryote toxin-antitoxin modules: complex regulation of an unclear function. Protein Sci.

[56] Kurata T, Kumar Saha C, Buttress JA, Mets T, Brodiazhenko T, Turnbull KJ, et al. A hyperpromiscuous antitoxin protein domain for the neutralization of diverse toxin domains. Proc Natl Acad Sci 2022;119:e2102212119. 10.1073/pnas.2102212119 PMC8832971 35121656

[57] Amitai S, Kolodkin-Gal I, Hananya-Meltabashi M, Sacher A, Engelberg-Kulka H (2009). *Escherichia coli* MazF leads to the simultaneous selective synthesis of both “death proteins” and “survival proteins. PloS Genet.

[58] Gerdes K, Christensen SK, Lobner-Olesen A (2005). Prokaryotic toxin-antitoxin stress response loci. Nat Rev Microbiol.

[59] Burroughs AM, Zhang D, Schaffer DE, Iyer LM, Aravind L (2015). Comparative genomic analyses reveal a vast, novel network of nucleotide-centric systems in biological conflicts, immunity and signalling. Nucleic Acids Res.

[60] Koonin EV, Makarova K (2019). Origins and evolution of CRISPR-Cas systems. Philos Trans R Soc B.

[61] Taylor D, Schwartz E, Bravo JPK, Ahsan M, Macias LA, McCafferty CL, et al. Type III CRISPR-Cas effectors act as protein-assisted ribozymes during RNA cleavage. Res. Sq. 2023. 10.21203/rs.3.rs-2837968/v1.

[62] Makarova K, Wolf YI, Alkhnbashi OS, Costa F, Shah SA, Saunders SJ (2015). An updated evolutionary classification of CRISPR-Cas systems. Nat Rev Microbiol.

[63] Mohanraju P, Saha C, van Baarlen P, Louwen R, Staals RHJ, van der Oost J (2022). Alternative functions of CRISPR-Cas systems in the evolutionary arms race. Nat Rev Microbiol.

[64] Garcia-Doval C, Schwede F, Berk C, Rostol JT, Niewoehner O, Tejero O (2020). Activation and self-inactivation mechanisms of the cyclic oligoadenylate-dependent CRISPR ribonuclease Csm6. Nat Commun.

[65] Sorokin DY, Tourova TP, Mussman M, Muyzer G (2008). *Dethiobacter alkaliphilus* gen. nov. sp. nov., and *Desulfurivibrio alkaliphilus* gen. nov. sp. nov.: two novel representatives of reductive sulfur cycle from soda lakes. Extremophiles.

[66] Melton ED, Sorokin DY, Overmars L, Lapidus AL, Pillay M, Ivanova N (2017). Draft genome sequence of *Dethiobacter alkaliphilus* strain AHT1^T^, a gram-positive sufidogenic polyextremophile. Stand Genom Sci.

[67] Zhilina TN, Zavarzin GA, Rainey FA, Pikuta EN, Osipov GA, Kostrikina NA (1997). *Desulfonatronovibrio hydrogenovorans* gen. nov., sp. nov., an alkaliphilic, sulfate-reducing bacterium. Int J Syst Evol Microbiol.

[68] Heyer H, Stal L, Krumbein WE (1989). Simultaneous heterolactic and acetate fermentation in the marine cyanobacterium *Oscillatoria limosa* incubated anaerobically in the dark. Arch Microbiol.

[69] Lee JZ, Burow LC, Woebken D, Everroad RC, Kubo MD, Spormann AM (2014). Fermentation couples *Chloroflexi* and sulfate-reducing bacteria to *Cyanobacteria* in hypersaline microbial mats. Front Microbiol.

[70] Stal LJ, Moezelaar R (1997). Fermentation in Cyanobacteria. FEMS Microbiol Rev.

[71] Ndhlovu A, Durand PM, Ramsey G (2020). Programmed cell death as a black queen in microbial communities. Mol Ecol.

[72] Refardt D, Bergmiller T, Kummerli R (2013). Altruism can evolve when relatedness is low: evidence from bacteria committing suicide upon phage infection. Proc R Soc B: Biol Sci.

[73] Orellana MV, Pang WL, Durand PM, Whitehead K, Baliga NS (2013). A role for programmed cell death in the microbial loop. PLoS One.

[74] Durand PM, Choudhury R, Rashidi A, Michod RE (2014). Programmed death in a unicellular organism has species-specific fitness effects. Biol Lett.

[75] Bar-Zeev E, Avishay I, Bidle KD, Berman-Frank I (2013). Programmed cell death in the marine cyanobacterium *Trichodesmium* mediates carbon and nitrogen export. ISME J.

[76] Ross C, Santiago-Vazquez, Paul V (2006). Toxin release in response to oxidative stress and programmed cell death in the cyanobacterium *Microcystis aeruginosa*. Aquat Toxicol.

[77] Richardson LL, Castenholz RW (1987). Enhanced survival of the cyanobacterium *Oscillatoria terebriformis* in darkness under anaerobic conditions. Appl Environ Microbiol.

[78] Jacobson MD (1996). Reactive oxygen species and programmed cell death. Trends Biochem Sci.

[79] Hong Y, Zeng J, Wang X, Zhao X (2019). Post-stress bacterial cell death mediated by reactive oxygen species. Proc Natl Acad Sci.

[80] Zhao X, Drlica K (2014). Reactive oxygen species and the bacterial response to lethal stress. Curr Opin Microbiol.

[81] Van Acker H, Coenye T (2017). The role of reactive oxygen species in antibiotic-mediated killing of bacteria. Trends Microbiol.

[82] Vincent RK, Qin X, McKay RML, Miner J, Czajkowski K, Savino J (2004). Phycocyanin detection from LANDSAT TM data for mapping cyanobacterial blooms in Lake Erie. Remote Sens Environ.

[83] Kupferschmidt K (2019). In search of blue. Science.

[84] Ataeian M, Liu Y, Canon-Rubio K, Nightingale M, Strous M, Vadlamani A (2019). Direct capture and conversion of CO2 from air by growing a cyanobacterial consortium at pH up to 11.2. Biotechnol Bioeng.

[85] Van Wychen S, Laurens LM. Determination of total carbohydrates in algal biomass: Laboratory analytical procedure (LAP). *National Renewable Energy Lab*. (2016).

[86] Novotnik B, Zorz J, Bryant S, Strous M (2019). The effect of dissimilatory manganese reduction on lactate fermentation and microbial community assembly. Front Microbiol.

[87] Patil G, Chethana S, Sridevi AS, Raghavarao KSMS (2006). Method to obtain C-phycocyanin of high purity. J Chromatogr A.

[88] Rinke C, Lee J, Nath N, Goudeau D, Thompson B, Poulton N (2014). Obtaining genomes from uncultivated environmental microorganisms using FACS-based single-cell genomics. Nat Protoc.

[89] Stoeck T, Bass D, Nebel M, Christen R, Jones MDM, Breiner H-W (2010). Multiple marker parallel tag environmental DNA sequencing reveals a highly complex eukaryotic community in marine anoxic water. Mol Ecol.

[90] Dong X, Kleiner M, Sharp CE, Thorson E, Li C, Liu D (2017). Fast and simple analysis of MiSeq amplicon sequencing data with MetaAmp. Front Microbiol.

[91] Quast C, Pruesse E, Yilmaz P, Gerken J, Schweer T, Yarza P (2013). The SILVA ribosomal RNA gene database project: improved data processing and web-based tools. Nucl Acids Res.

[92] Paquette AJ, Sharp CE, Schnurr JP, Allen DG, Short SM, Espie GS (2020). Dynamic changes in community composition of *Scenedesmus*-seeded artificial, engineered microalgal biofilms. Algal Res.

[93] Bushnell B, Rood J, Singer E (2017). BBMerge - Accurate paired shotgun read merging via overlap. PloS One.

[94] Nurk S, Meleshko D, Korobeynikov A, Pevzner PA (2017). metaSPAdes: a new versatile metagenomic assembler. Genome Res.

[95] Li D, Liu C-M, Luo R, Sadakane K, Lam T-W (2015). MEGAHIT: an ultra-fast single-node solution for large and complex metagenomics assembly via succinct *de Bruijn* graph. Bioinformatics.

[96] Dong X, Strous M (2019). An integrated pipeline for annotation and visualization of metagenomic contigs. Front Genet.

[97] Kang DD, Li F, Kirton E, Thomas A, Egan R, An H (2019). MetaBAT2: an adaptive binning algorithm for robust and efficient genome reconstruction from metagenome assemblies. PeerJ.

[98] Kang D, Froula J, Egan R, Wang Z (2015). MetaBAT, an efficient tool for accurately reconstructing single genomes from complex microbial communities. PeerJ.

[99] Olm MR, Brown CT, Brooks B, Banfield JF (2017). dRep: a tool for fast and accurate genomic comparisons that enables improved genome recovery from metagenomes through de-replication. ISME J.

[100] Parks D, Imelfort M, Skennerton CT, Hugenholtz P, Tyson GW (2015). CheckM: assessing the quality of microbial genomes recovered from isolates, single cells, and metagenomes. Genome Res.

[101] Chaumeil P-A, Mussig AJ, Hugenholtz P, Parks DH (2020). GTDB-Tk: a toolkit to classify genomes with the Genome Taxonomy Database. Bioinformatics.

[102] Li W, Godzik A (2006). Cd-hit: a fast program for clustering and comparing large sets of protein or nucleotide sequences. Bioinformatics.

[103] Gruber-Vodicka HR, Seah BKB, Pruesse E (2020). phyloFlash: Rapid small-subunit rRNA profiling and targeted assembly from metagenomes. mSystems.

[104] McLeod AI. The Kendall package. *CRAN*. (2005). http://www.stats.uwo.ca/faculty/aim.

[105] Kleiner M, Thorson E, Sharp CE, Dong X, Liu D, Li C (2017). Assessing species biomass contributions in microbial communities via metaproteomics. Nat Commun.

[106] Wisniewski JR, Zougman A, Nagaraj N, Mann M (2009). Universal sample preparation method for proteome analysis. Nat Meth.

[107] Hinzke T, Kouris A, Hughes R-A, Strous M, Kleiner M (2019). More is not always better: evaluation of 1D and 2D-LC-MS/MS Methods for metaproteomics. Front Microbiol.

[108] Petersen JM, Kemper A, Gruber-Vodicka H, Cardini U, van der Geest M, Kleiner M (2016). Chemosynthetic symbionts of marine invertebrate animals are capable of nitrogen fixation. Nat Microbiol.

[109] Zybailov B, Mosley AL, Sardiu ME, Coleman MK, Florens L, Washburn MP (2006). Statistical analysis of membrane proteome expression changes in *Saccharomyces cerevisiae*. J Proteome Res.

[110] Dixon P (2003). VEGAN, a package of R functions for community ecology. J Veg Sci.

[111] De Caceres M, Legendre P, Moretti M (2010). Improving indicator species analysis by combining groups of sites. Oikos.

[112] Barnes RJ, Voegtlin SP, Hubert CRJ, Larter SR, Bryant SL (2022). The critical role of environmental synergies in the creation of bionanohybrid microbes. Appl Environ Microbiol.

