# Supplementary material for: Coordinated proteome change precedes cell lysis and death in a mat-forming cyanobacterium
Source: ISME J. 2023 Nov 1;17(12):2403–14. doi: 10.1038/s41396-023-01545-3 (PMC10689466; doi:10.1038/s41396-023-01545-3)
Supplement: Supplementary file 1 — Supplementary Material [file 41396_2023_1545_MOESM1_ESM.pdf]

Supplementary Materials for

Coordinated proteome change precedes cell lysis and death in a mat-forming cyanobacterium

Jackie Zorz*, Alex Paquette, Timber Gillis, Angela Kouris, Varada Khot, Cigdem Demirkaya, Hector De La Hoz Siegler, Marc Strous, Agasteswar Vadlamani

*Corresponding author. Email: jacqueline.zorz@ucalgary.ca

This file includes:

Figs. S1 to S6

Other supplementary materials for this manuscript include the following:

Tables S1 to S3
Supplementary Table 1 - MAG information
Supplementary Table 2 - Metaproteome protein abundances
Supplementary Table 3 – Proteins identified as significantly associated with an incubation fraction

Data S1
Supplementary Data 1 - Predicted proteins and corresponding accessions used in this manuscript from the Ca. P. alkaliphilum MAG

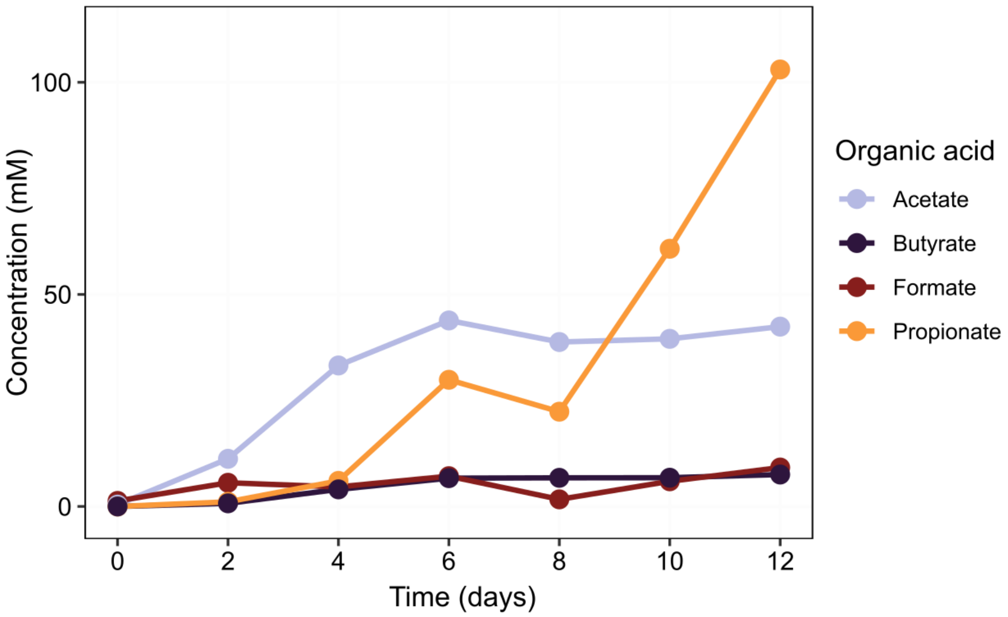

Fig. S1.
Concentrations of organic acids in the supernatant fraction during the incubation. Succinate and formate were also measured but not detected during the incubation.

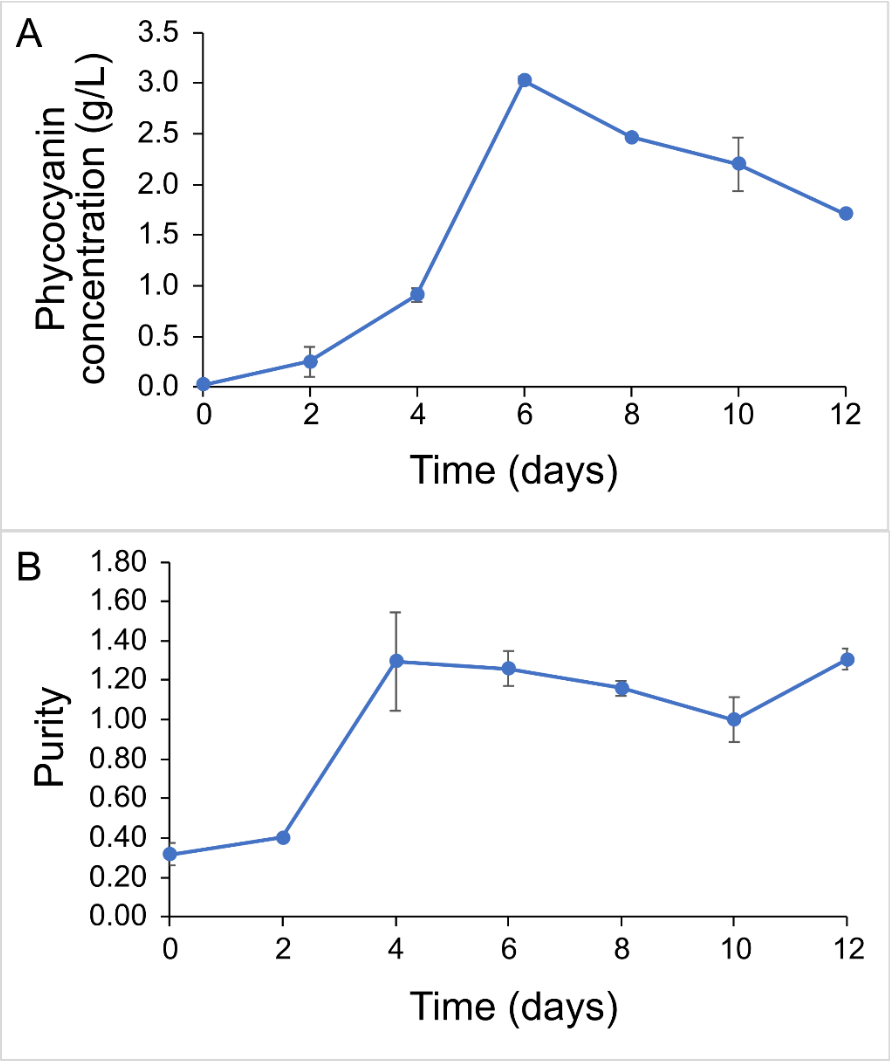

Fig. S2.
Concentration and purity of released phycocyanin during the incubation. (A) Phycocyanin concentration measured as absorbance at 620 nm compared to a standard curve of laboratory grade phycocyanin. (B) Purity was measured as the absorbance ratio of 620/280 nm.

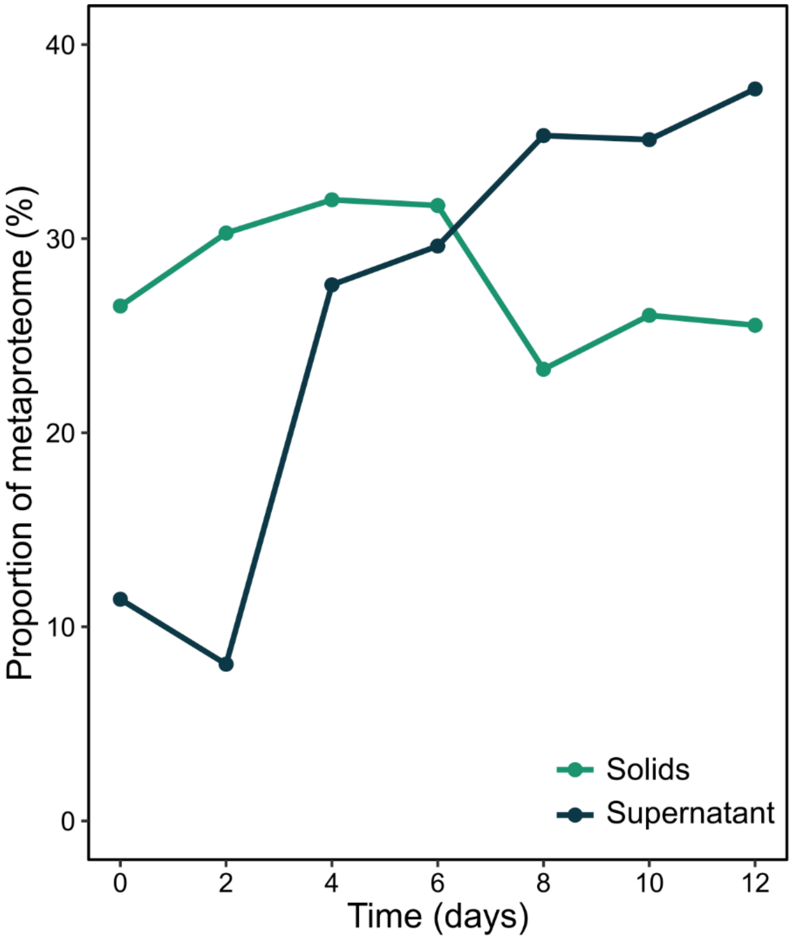

Fig. S3.
Relative abundance of *Ca.* P. alkaliphilum pigment proteins in the metaproteome during dark and anoxic incubation. Proteins from the solid fraction are shown in green and proteins from the supernatant fraction are shown in dark blue.

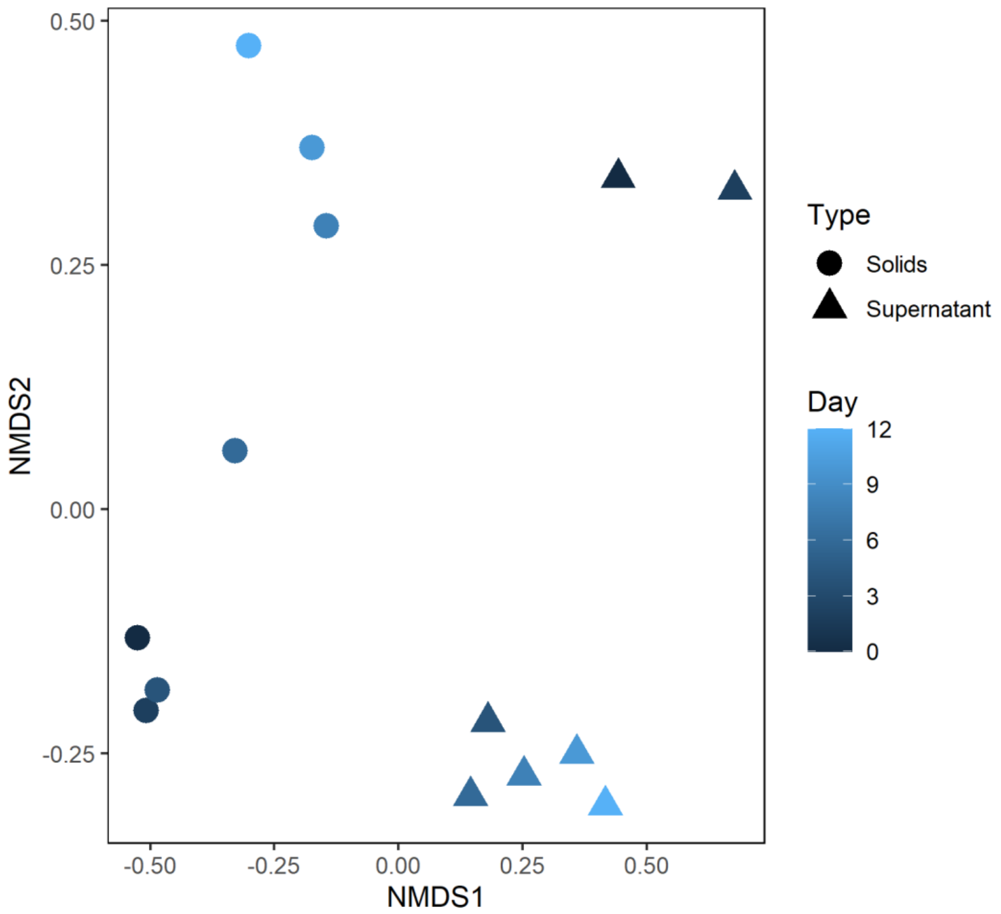

Fig S4.
NMDS of incubation samples based on Bray-Curtis dissimilarity of protein abundance. Only proteins with more than 50 PSMs included. Stress = 0.09.

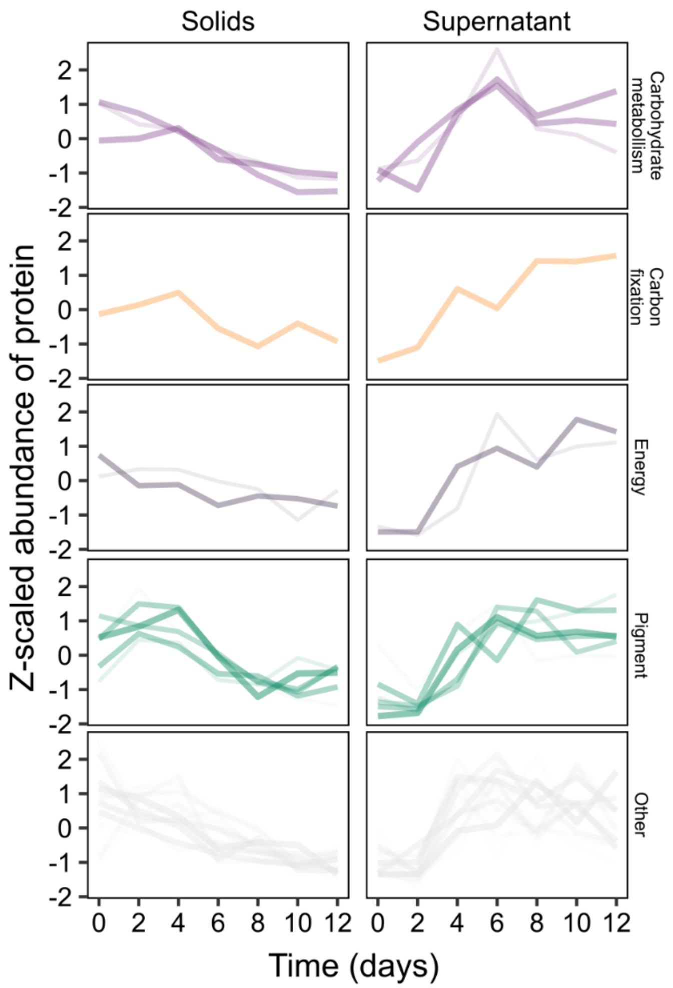

Fig S5.
Proteins from functional categories that were significantly associated with both pre-lysis solids and post-lysis supernatant fractions. These proteins were likely intracellular proteins released into the media upon cell lysis. Protein abundances were scaled across samples for visualization purposes. For a complete list of these proteins see Supplementary Table 3.

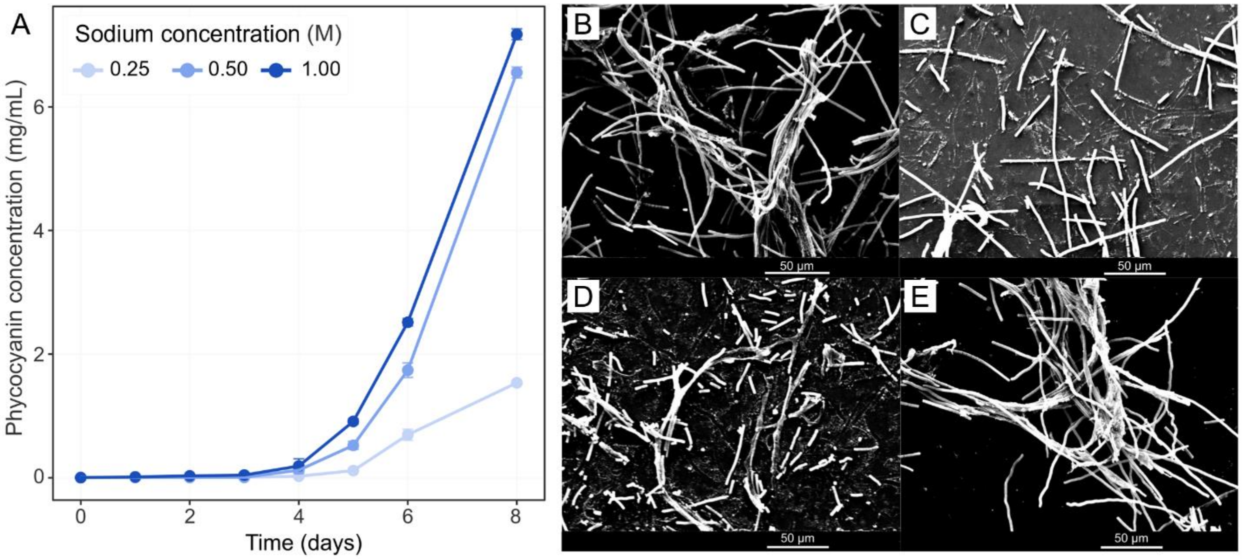

Fig S6.
Dark and anoxic incubations with varying sodium concentration. (A) Phycocyanin concentration in the supernatant fraction of dark and anoxic incubations with varying sodium concentrations in the media. Electron micrographs of cyanobacterial cells on day 0 (B), and day 5 of dark and anoxic incubations, with the original 0.5 M Na^+ media (C), 1 M Na^+ media (D), and 0.25 M Na^+ media (E).

Table S1. (separate file)
MAG information. Contains information on all metagenome assembled genomes present in the consortium.

Table S2. (separate file)
Metaproteome protein abundances. Contains abundances of all proteins identified in the metaproteomes.

Table S3. (separate file)
Proteins identified as significantly associated with an incubation fraction.

Data S1. (separate file)
Predicted proteins and corresponding accessions used in this manuscript from the *Ca.* P. alkaliphilum MAG with annotations from MetaErg.
